# Practice and Enlightenment of Chronic Disease Management at the County Level in China from the Perspective of Professional Integration: A Qualitative Case Study of Youxi County, Fujian Province

**DOI:** 10.5334/ijic.7550

**Published:** 2023-08-10

**Authors:** Ying Zheng, Jia Hu, Li Li, Tao Dai

**Affiliations:** 1 Center for Health Policy and Management, Institute of Medical Information & Library, Chinese Academy of Medical Sciences & Peking Union Medical College, Beijing 100020, CN; 2 Center for Health Policy and Management, Chinese Academy of Medical Sciences & Peking Union Medical College, Beijing, 100020, CN

**Keywords:** professional integration, chronic disease management, qualitative case study, county, China

## Abstract

**Background:**

It is currently the most cost-effective management model to have multiple professionals from relevant institutions collaborate so as to provide integrated chronic disease management services. The “classified, color-coded, hierarchical and regionalized” chronic disease management model in Youxi County, Fujian Province is a typical case in China. However, related research is limited. This paper aims to analyze the practice measures and lessons learned in Youxi County, focusing on the professional integration of service providers.

**Methods:**

From January to March 2021, interviews with 15 key informants in Youxi County were conducted to collect qualitative data, which was analyzed by the thematic framework method as well as the policy data, using the professional integration dimension in the evaluation framework of the integrated healthcare system.

**Results:**

A series of measures were taken, such as improving the professional division and collaboration mechanism, establishing the incentive and restraint mechanism geared toward chronic disease management, formulating norms and standards of chronic disease management for patients with different color labels, and promoting the compatibility of inter-professional value and culture under the governmental institutional supply and the organizational support of the tight county healthcare alliance in Youxi County, to prompt professionals of different levels and types to collaborate in order to provide integrated chronic disease management services. However, some problems remained, such as limited capacity of primary health care, the relatively narrow range and weak effect of the incentive and restraint mechanism, inadequate implementation of the norms and standards, and so forth.

**Conclusions:**

Our findings provide reference for other regions in China and other low- and middle-income countries in exploring the integrated chronic disease management model. Long-term follow-up surveys and mixed research designs are required in the future to enrich relevant evidence.

## (1) Introduction

Chronic non-communicable diseases (hereinafter referred to as “chronic diseases”) have become an important factor affecting global people’s health and the leading cause of premature death and disability, mostly in low- and middle-income countries. This situation aggravates the health inequity and affects the achievement of Universal Health Coverage and the Sustainable Development Goals. According to statistics, the global proportion of deaths caused by chronic diseases increased from 60.8% in 2000 to 73.6% in 2019, with low- and middle-income countries accounting for approximately 77% of deaths and 85% of premature deaths caused by chronic diseases [1,2]. While in China, the proportion of deaths caused by chronic diseases was 88.5% in 2019, significantly higher than the global averages, and the premature mortality rate of major chronic diseases (cardiovascular and cerebrovascular diseases, cancer, chronic respiratory diseases and diabetes) was 16.5%, higher than the Western Pacific regions, European and North American averages (15.6%, 16.3% and 14.0% respectively) [1,2,3]. The high mortality rates and premature mortality rates highlight the importance and urgency of chronic disease management, and it is extremely critical for any country to raise awareness and investment in it so as to protect its people’s health [4].

The pathogenic factors of chronic diseases are complicated. The patients are always difficult to be detected in the early stage as well as easy to relapse, and have a long disease course and multiple comorbidities. Relevant research and practice have shown that it is currently the most cost-effective chronic disease management model to have multiple professionals from relevant institutions collaborate in order to provide comprehensive, whole-process, whole-life-cycle, and integrated chronic disease management services for patients, covering prevention, treatment, rehabilitation, and health promotion from hospitals, communities and families [5,6,7]. This has been proved by the models proposed by foreign scholars such as the Chronic Care Model (CCM) [8], the Innovative Care for Chronic Conditions Framework (ICCC Framework) [9], the Guided Care Model (GCM) [10], the SELFIE framework for integrated care for multi-morbidity [11] and the ones proposed by Chinese scholars such as Xiamen City’s “Specialty Physician-General Practitioner-Health Manager” Chronic Disease Management Model [12], the “Hospital-Community-Patient-Volunteer” Integrated Chronic Disease Management Model [13], and the “County-level Hospital-County-level CDC-Township Hospital, Community-Family” Integrated Chronic Disease Management Model [14]. According to our previous research results, professional integration is an important dimension to analyze integrated health care, which focuses on the key elements of collaboration among service providers at the micro level [15,16]. Research on the integrated chronic disease management model from the above perspective, that is, analyzing the collaboration mechanism among different professionals in the same institution or different institutions involved in chronic disease management, is conducive to an in-depth analysis of the key elements of the model at the micro level and the discovery of deficiencies to optimize and improve the chronic disease management services.

Since the new health care system reform, China has issued a series of special policies concerning chronic diseases, including “China’s chronic disease prevention and control work plan (2012–2015),” “China’s medium- and long-term plan for chronic disease prevention and control (2017–2025),” “Guidance on improving the medication guarantee mechanism of hypertension and diabetes outpatients in urban and rural residents,” and so forth. It has also been included in the Healthy China Strategy. Meanwhile, China has been constructing the comprehensive prevention and control demonstration zone of chronic diseases in county-level administrative division since 2010, in conjunction with the county healthcare alliance reform, which has made excellent progress. Thereinto, the “classified, color-coded, hierarchical and regionalized” chronic disease management model pioneered by Youxi County, Fujian Province in June 2018, in which relevant professionals in health institutions of different levels and types would take charge of the management of the patients with different types of chronic diseases who are color-coded according to their disease conditions in corresponding regions, has received positive evaluations from the World Bank, the State Council and the National Health Commission of the People’s Republic of China, China’s relevant industry associations, and so on [17,18]. However, the related research is still not rich. Existing research either briefly introduced it as part of the case analysis of the tight county healthcare alliance reform in Youxi County, or analyzed the effects of chronic disease management from the perspective of demanders such as patients’ health behaviors and health outcomes, while lacked in-depth analysis of specific measures of the chronic disease management from the perspective of suppliers. [19,20,21,22] Hence, this paper intends to conduct an in-depth analysis of the professional collaboration mechanism of the chronic disease management model in Youxi County based on the professional integration dimension in the evaluation framework of the integrated healthcare system (hereinafter referred to as “IHS evaluation framework”) developed by our team [16,23], summarize the practical lessons, discover problems and put forward policy recommendations, to provide reference for the chronic disease management practice in China and other low- and middle-income countries.

## (2) Methods

### (2.1) Case selection

Considering the typicality and representativeness, Youxi County was selected to conduct this qualitative case study. On one hand, Youxi County has a good foundation for reform as the pilot area of Sanming City, Fujian Province, which is China’s health care system reform model. It was identified as one of the first national demonstration counties of comprehensive reform of public hospitals in 2012, and took the lead in conducting the comprehensive reform of “medical treatment, medical insurance, and medicine linkage” (hereinafter referred to as “three medical linkage reform”), which has been widely promoted in mainland China. Then it took the tight county healthcare alliance as a carrier to build a people-centered integrated healthcare system in April 2017. On this basis, it began to explore the “classified, color-coded, hierarchical and regionalized” chronic disease management model in June 2018 which had been about three years up to the date of investigation. On the other hand, the practice of chronic disease management in Youxi County has achieved good results. For example, the blood pressure control rate of the hypertension management population and the blood glucose control rate of the diabetes management population were 85.45% and 84.83% in 2020, which were 19.65 and 19.82 percentage points higher than those before the reform respectively. The practice had been promoted in mainland China as Sanming City’s experience of deepening health care system reform since November 2019 and has received multiple positive evaluation. Youxi County was also selected as the provincial comprehensive prevention and control demonstration zone of chronic diseases in 2020.

### (2.2) Analytical framework

The professional integration dimension in the IHS evaluation framework developed by our team was used as the analytical framework, mainly including four elements: professional division and collaboration, incentive and restraint mechanism, inter-professional service norms and standards, and inter-professional compatible value and culture (see Table 1 for details). [16,23] Based on the Rainbow Model of Integrated Care (RMIC) which has been highly recognized by researchers in different countries, our team used systematic review, thematic framework method and two Delphi studies to develop the IHS evaluation framework accordance with China’s actual conditions. The professional integration dimension of IHS evaluation framework had been validated in the county healthcare alliances of Tianchang City, Anhui Province, which confirmed its adaptation and applicability in China [24]. Therefore, it is appropriate to take the professional integration dimension in the IHS evaluation framework as our analytical framework to analyze the chronic disease management model in the tight county healthcare alliances in Youxi County.

**Table 1:** The analytical framework of the “classified, color-coded, hierarchical and regionalized” chronic disease management model of Youxi County from the perspective of professional integration.


ELEMENTS	CONNOTATION

professional division and collaboration	inter-professional coordination mechanisms, forms and tightness degree of inter-professional collaboration

incentive and restraint mechanism	incentive and restraint mechanisms of division and collaboration among different professionals

inter-professional service norms and standards	formulating targeted, instructive and standardized inter-professional service norms and standards

inter-professional compatible value and culture	shared value of “people-centered” and trust among different professionals


### (2.3) Data collection

The qualitative data including policy documents and interviews was collected from January to March in 2021. To obtain a full understanding of the professional integration measures of chronic disease management model in Youxi County, the interviewees included the policymakers and implementers of chronic disease management. Interviewees were recruited until information saturation was reached. The final number of interviewees was fifteen(three staff of the County Health Bureau, two administrative staff and three health professionals of the County General Hospital, three staff of the County Center for Disease Control and Prevention (hereinafter referred to as “CDC”), one administrative staff and two health professionals of the township hospitals, and one rural doctor). Individual semi-structured interviews were conducted and were recorded to guarantee the authenticity and integrity of the content after acquiring their informed consent. The average time of each interview was one hour. The interview outline was designed based on the analytical framework focusing on specific measures and problems of the chronic disease management in Youxi county from the four elements, including the following: the division of different professionals’ responsibilities, inter-professional coordination mechanisms, forms and tightness degree of inter-professional collaboration, incentive and restraint mechanisms such as compensation distribution and performance appraisal, inter-professional service norms and standards, the value they believe in and their trust in other professionals, etc. In addition, policy documents about chronic disease management were gathered through the County Health Bureau’s provision of relevant staff and retrieval of government portal websites such as the Secretariat of the Leading Group of Deepening the Health Care System Reform of Sanming City, the Healthcare Security Administration of Sanming City, and so forth.

### (2.4) Data analysis

The interview recordings were transcribed into text and cross-examined by two researchers. The thematic framework method was used to sort out and analyze these qualitative data based on the analytical framework shown in Table 1. Specifically, the four elements of professional integration was used as the basic framework(deductive). Open labels (inductive) developed by two researchers were used to identify relevant themes that emerged under each element. Excel 2016 was used to process the data.In the process of theme induction, we compared the interviews of different stakeholders and policy documents to ensure the authenticity and reliability of data analysis.

## (3) Results

### (3.1) Practice context and general thoughts

**Figure 1:**
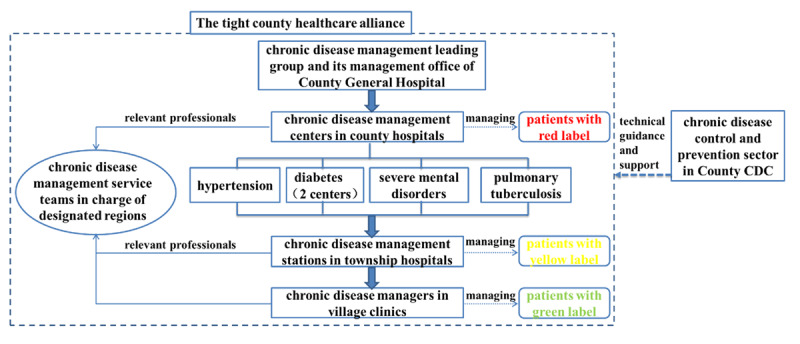
“Classified, color-coded, hierarchical and regionalized” chronic disease management model of Youxi County.

In order to implement the guidelines for health work of “prevention first, prevention and treatment combined” and the strategic requirements of “Healthy China”, and to address the increasing burden of chronic diseases effectively owing to accelerated population aging and lifestyles changes, Youxi County, based on the three medical linkage reform and the construction of tight county healthcare alliance(integrating the county hospitals, township hospitals and village clinics to establish the County General Hospital with one independent legal personality),selected four types of the main chronic diseases such as hypertension, diabetes, severe mental disorders and pulmonary tuberculosis (classified management); divided the patients into different groups with different color labels according to their disease conditions (color-coded management), and clarified the responsibilities and responsible regions of relevant professionals in health institutions of different levels and types (hierarchical and regionalized management) in order to explore the “classified, color-coded, hierarchical and regionalized” chronic disease management model in June 2018 (see Figure 1). Meanwhile, Youxi County established leading organizations headed by principle leaders of county party committee and county government in which all relevant departments leaders all participated in, such as the leading group for building the provincial comprehensive prevention and control demonstration zone of chronic diseases, the leading group for the health responsibility system and the leading group for the integration of medical treatment and prevention. It also set up special funds that were included in the financial annual budget and final accounts to create synergy for promoting the exploration and innovation of the chronic disease management model. Through the implementation of the management model, Youxi County strove to provide comprehensive, whole-process and whole-lifecycle integrated chronic disease management services for the county residents.

### (3.2) Division and collaboration

The professionals involved in the chronic disease management model in Youxi County mainly included the medical staff of relevant clinical specialties in the county hospitals and township hospitals, the rural doctors in village clinics, and the chronic disease control and prevention personnel in county CDC. A series of measures were taken to promote “hierarchical and regionalized” management, such as clarifying the division of responsibilities, strengthening communication and coordination, enriching collaboration forms, and so forth.

Firstly, clarifying the division of different professionals’ responsibilities. Five chronic disease management centers were established in two county hospitals (four centers in County General Hospital including hypertension, diabetes, severe mental disorders, and pulmonary tuberculosis; and one diabetes center in County Traditional Chinese Medicine Hospital, hereinafter referred to as “county center”), with directors and deputy directors served by the chief physicians of relevant clinical sectors. The professionals in county centers were in charge of negotiating with those in township hospitals to formulate the management plans, norms, standards and regulations; organizing large-scale health education and screening activities of chronic diseases to screen out new patients who didn’t have health records and were not listed in the management information system of primary healthcare institutions (hereinafter referred to as “primary healthcare information system”) and hence, sending their information to the professionals in the chronic disease management stations of township hospitals where they lived; providing standardized treatment and health management services for red label patients, adjusting the color code according to disease conditions as well as making information referral in time; conducting technical guidance, training, quality control, supervision and assessment of chronic disease management for those in primary healthcare institutions. A chronic disease management station was set up in each township hospital (hereinafter referred to as “township station”), and the professionals there had the following responsibilities: conducting health education, establishing health records, registering outpatient special diseases and providing standardized management services for new patients sent from county centers and detected through first diagnosis, health examination, health education activities, and so forth; providing standardized treatment and health management services for yellow label patients, adjusting the color code according to disease conditions and making information referral in time; conducting training and technical guidance for chronic disease managers in village clinics. The rural doctor in each village clinic served as chronic disease manager and was responsible for providing daily health management services for green label patients as well as adjusting the color code according to disease conditions and making information referral in time. Moreover, a chronic disease control and prevention sector was set up in County CDC (hereinafter referred to as “CDC sector”) and was staffed with full-time professionals who were responsible for providing technical guidance and support to those working in county hospitals, township hospitals, and village clinics.

Secondly, establishing and improving communication and coordination mechanisms among professionals. A chronic disease management leading group was established. The Party Secretary of County General Hospital was appointed as the leader, the hospital’s director and vice director as deputy leaders, and the heads of other institutions in the county healthcare alliance as members. A management office of the leading group was established to handle communication and coordination issues across institutions, allowing different professionals to participate in the decision-making. The county centers, township stations, and CDC sector all had dedicated staff as coordinators to facilitate communication and coordination among different institutions and professionals. Information technology was used to unblock inter-professional communication channels and improve management and service efficiency. For example, an integrated chronic disease management information platform, a two-way referral information platform, the APP of “medical service cloud” for medical staff, and the APP of “personal health cloud” for patients were developed and interconnected with each other as well as the primary healthcare information system; WeChat groups of chronic disease management teams were established to enable different professionals and patients interact online and gain access to health information at anytime and anywhere; artificial intelligence voice technology was also used to carry out follow-ups, satisfaction surveys and health education for discharged patients.

“The Party Secretary of our hospital bears the main responsibility. A specialized chronic disease management office was set up. The center has dedicated staff responsible for communication and coordination, and a specialized information platform and many mobile phone software were developed. The communication between the professionals of the member units and us is much more convenient than before.” [County General Hospital administrative staff]

Thirdly, carrying out various forms of professional collaboration. Ninety chronic disease management service teams were established consisting of medical staff from county, township and village healthcare institutions. Each team was in charge of a designated region based on the administrative divisions and the number of patients with chronic diseases. In addition, professionals in township hospitals and village clinics were in charge of daily health management and collaborated with the specialists in county hospitals who worked at primary healthcare institutions regularly to provide standardized treatment for patients with chronic diseases. Experts from county centers and CDC sector provided chronic disease management training to professionals from primary healthcare institutions at least once a quarter. Professionals from primary healthcare institutions would also be selected to attend county hospitals for further study.

“I am responsible for conducting daily health management for the patients with chronic diseases in our village. The (chronic disease management service) team members also include doctors from our superior township hospital and the County General Hospital. If I have any questions, I can ask them at any time. They will also come here for guidance regularly or carry out remote training, which do help me a lot with my capacity improvement.” [Rural doctor]

However, the majority of interviewees indicated that medical staff in county hospitals continued to take the lead, whether in clarifying the division of responsibilities or in the chronic disease management practice, whereas the core role of professionals in primary healthcare institutions in chronic disease screening, risk assessment, health guidance and other aspects had not been effectively played. Chronic disease management led by medical staff in county hospitals might lack sustainability due to intensive clinical tasks.

“Owing to the poor capacity of primary health care, the chronic disease management of the whole county is still led by medical staff in county hospitals, including formulating norms and conducting screening, risk assessment, treatment, and so forth; while those in primary healthcare institutions are just responsible for simple health management.” [County Health Bureau staff]

### (3.3) Incentive and restraint mechanism

Mechanisms geared toward chronic disease management were established to form external incentives and restraints for different professionals to enhance their enthusiasm so as to provide collaborative chronic disease management services, including compensation distribution, performance appraisal, professional title promotion, and so forth.

Firstly, based on the annual salary work-point system of medical staff in county hospitals and the performance pay work-point system of those in primary healthcare institutions, special work-points for chronic disease management services and basic public health services were established to facilitate professionals in county and township hospitals earn more work-points and thus more compensation through the supply of chronic disease management services. For example, professionals establishing a health record for a hypertensive patient could earn 5 work-points; providing a follow-up service or a personalized health guidance service for a patient could earn 15 or 5 work-points, and so forth.

Secondly, a performance appraisal bonus for integrated chronic disease management was established. The performance appraisal system consisted of three first grade indexes (organization management, health management, and health outcome), 11 second grade indexes, and 33 third grade indexes; furthermore, there were extra bonus points reflecting innovation and highlights, and penalty deduction points reflecting quality and authenticity. The performance appraisal was conducted at the city level. The amount of the bonus, which was determined by Youxi County’s ranking in the city, would be incorporated into the total wages of professionals and then distributed by County General Hospital according to the work-point system.

Meanwhile, a series of measures were implemented to encourage the initiative of professionals from county hospitals to serve in primary healthcare institutions and provide technical assistance and guidance for professionals there, creating opportunities for professionals at different levels to carry out technical collaboration on chronic disease management, such as implementing the policy of serving for more than one year in primary healthcare institutions prior to promotion of professional titles, adjusting the work-point value of 11 diseases in county hospitals (lowering the value) and township hospitals (raising the value), and so forth. In addition, the welfare of rural doctors was improved to a certain extent in order to arouse their enthusiasm for providing chronic disease management services as quasi-public products, through measures such as dispensing subsidy, establishing the pension insurance subsidy system, and implementing a new personal management system for rural doctors (managed by county hospitals, employed by township hospitals, and used by village clinics), and so forth.

However, the county CDC’s incentives for chronic disease control and prevention personnel were still deficient. The fact that their salary was far lower than that of medical staff at the same level might have dampened their enthusiasm to participate in inter-professional collaboration to a certain extent. According to the interview, the proportion of performance-based salary received by professionals in county hospitals for providing chronic disease management services was not high enough to form an effective incentive and restraint effect.

“Because of the special work-points and performance bonus for chronic disease management services, our motivation do improve a lot than before. However, their proportion is extremely low compared to the treatment services. So, we may ignore them when we are busy with the heavy clinical treatment task.” [County General Hospital doctor]

“Now we can get subsidies for cost-of-living every month and for pension insurance from the county financial department, and are also under the unified management of township hospital, which make our welfare and social status improve a lot. So we are more motivated to complete the task of chronic disease management.” [Rural doctor]

### (3.4) Inter-Professional service norms and standards

Patients with chronic diseases in Youxi County were divided into different groups with different color labels according to their disease conditions referring to national standards, that is, green label indicated patients with the lightest disease condition, yellow in the middle, and red the most severe condition. Chronic disease management norms and standards for patients with different color labels were formulated and health records were established to promote the “classified and color-coded” management. Specifically, patients with green label were those who had normal indexes and no complications upon taking essential drugs and would be provided with daily health management services by the chronic disease managers in village clinics. Moreover, their label color would be changed into yellow and information would be sent to the professionals in township stations in case their indexes were getting abnormal, disease condition changes were difficult to control, or new complications and adverse reactions occurred. After receiving the information, professionals in township stations would check their medical record at once, contact them initiatively, make an appointment for the outpatient services or conduct family and telephone follow-up services, and provide services such as health consultation, adjustment of drugs, and rational drug use guidance according to disease conditions. Their labels color would be changed into green and information would be sent back to the chronic disease managers in village clinics if the indexes could be kept within a reasonable range after standardized treatment and management. Whereas their labels color would be changed into red, information would be sent to the professionals in county centers if their indexes could still not return to normal or new changes of disease conditions occurred such as new complications, and adverse reactions. After receiving the information, professionals in county centers would handle the case as mentioned above and provide emergency diagnosis, treatment, and standardized management services. Their labels color would be changed into yellow or green and information would be sent back to the professionals in township stations or the chronic disease managers in village clinics if the indexes could be kept within a reasonable range.

Nevertheless, the majority of the inter-professional chronic disease management norms and standards were guidance documents lacking associated supervision and reward and punishment measures, resulting in their frequent inadequate implementation. For example, doctors in county hospitals failed to adjust the patients’ color labels and refer them back to primary healthcare institutions on time.

“The documents issued by the County General Hospital have stipulated the standards for the management and referral of chronic disease patients with different color labels, which is very helpful for us to conduct the chronic disease management. However, they (doctors at county hospitals) do not refer the patients back to us after treatment according to the documents sometimes, and would not get any punishment.” [Township Hospital doctor]

### (3.5) Inter-Professional compatible value and culture

Various measures were implemented in Youxi County including enhancing propaganda and mobilization, giving economic and non-economic incentives and so forth, to prompt professionals at different levels and types so as to improve their common value identification and finally internalize them, such as people-oriented, demand-oriented, prevention first and combination of prevention and treatment, resulting in the change of behaviors from passive implementation of policies to active and voluntary provision of integrated chronic disease management services.

“Since the state has vigorously promoted the concept of “people-centered” and the high cost-effectiveness of prevention and paid much more attention to the assessment of “patient satisfaction” in recent years, we are willing and eager to invest more time and effort in chronic disease management services, listen to the needs and preferences of patients and take them as an important basis for formulating management plans, and pay more attention to health education to improve the patients’ ability of self-health management and participate in clinical decision-making.” [Doctors in County General Hospital and Township Hospital]

On the other hand, a relatively good trust relationship had been established among professionals involved in chronic disease management owing to the long-term professional collaboration and the county’s characteristics of acquaintance society. However, there was an asymmetry of trust that professionals in township hospitals and village clinics had a higher degree of trust and identification than those in county hospitals.

“The experts in county hospitals have strong healthcare service capability and their guidance does greatly improve our capability of chronic disease management. When there is a problem, I prefer to seek help from such experts the first time.” [Township Hospital doctor]

“Owing to their poor healthcare service capability, I do not trust the professionals in primary healthcare institutions and I am afraid to refer patients to them.” [County General Hospital doctor]

## (4) Discussion

### (4.1) Key elements to facilitate the professional integration of chronic disease management services

Firstly, an inter-professional service team or network with general practitioners as the core, a clear division of responsibilities as well as smooth communication and coordination are prerequisite for facilitating the professional integration of chronic disease management services. It is one of the key elements of integration by which community general practitioners play a core role in an inter-professional service team or network, especially for chronic disease patients with multiple health and social service needs [25,26,27,28]. Although their service capability was enhanced through the technical assistance of the county-level professionals, it was still relatively poor and the professionals in Youxi County’s primary healthcare institutions were in the subordinate position in chronic disease management, which is not conductive to the sustainability of professional integration; and it may be related to the difficulty in attracting and retaining talents in rural primary healthcare institutions caused by China’s unbalanced development between urban and rural areas, insufficient financial investment, lag in the development of the general practice education system, and so forth [29,30], which is also the general problem in other regions of China and most low- and middle-income countries that need to pay enough attention to the policy and practice level and take targeted measures. Additionally, a highly integrated service team or network usually has a clear division of responsibilities and sound communication mechanisms and makes full use of information technology [28,31,32,33,34,35]. The division of members’ responsibilities in chronic disease management teams was clarified based on the function and service capabilities of the institutions in Youxi County, which is conducive to solving the problems of disordered competition, inefficient collaboration and resource waste due to unclear or overlapping responsibilities in order to promote professional integration. Meanwhile, in Youxi County, a specialized coordination organization and full-time coordinators were established as well as an interconnected information platform, which not only improved the collaboration efficiency and the continuity and coordination of services, but also could contribute to enhancing trust among members through smooth communication, thereby further promoting teamwork and forming a virtuous circle. Since they could be completed within the healthcare institutions and have strong operability, the above measures could be replicated in other regions of China and most low- and middle- income countries.

Secondly, effective incentives and restraints serve as the external motivation to encourage professionals to collaborate in order to provide integrated chronic disease management services. Professionals’ extra labor to provide chronic disease management services was compensated by establishing special work-points and separate bonuses in Youxi County, which could form certain external economic incentives to make it possible to promote the achievement of the compatibility of interests among professionals with different interest demands. This is consistent with the conclusion drawn by existing research that “adequate financial compensation and appropriate payment methods are important incentive factors to promote collaborative services” [32,36,37]. On the other hand, the assessment on the normalization of health management process and its effects could also form certain restraints on professionals’ chronic disease management service behaviors to ensure service quality. However, the above measures need to be further improved due to the fact that they did not cover all professionals and the economic incentives were inadequate, which might affect the policy effects. This also suggests that other regions of China and low- and middle- income countries should pay attention to the coverage and intensity of the incentive and restraint mechanisms when replicating the measures of Youxi County, which means the mechanisms should cover all professionals involved in chronic disease management, and the proportion of economic incentives should be determined based on scientific calculation.

Thirdly, perfect inter-professional service norms and standards are an important foundation and reference for ensuring the quality of integrated chronic disease management services. The formulation and effective implementation of the unified as well as evidence-based inter-professional service norms and standards could lay the foundation for providing high-quality integrated chronic disease management services, which is also one of the key elements of integration [24,31,38]. The management norms and standards for chronic disease patients with different color labels, which were formulated in Youxi County based on the national standards, clearly defined the contents such as professionals’ responsibilities, therapeutic schedules, referral processes, and so forth. They standardized the diagnostic and treatment behaviors of relevant professionals especially those in primary healthcare institutions, ensured the homogenization of services, and enhanced the continuity and coordination of services. Nevertheless, due to a lack of supervision, reward, and punishment measures, the implementation effects of the norms and standards were influenced to a certain extent, which is consistent with practice in other regions of China [24,39]. This suggests that other regions of China and low- and middle- income countries not only need to develop inter-professional service norms and standards in chronic disease management practice, but also should pay more attention to the setting of supervision, reward and punishment measures to ensure the implementation of standards and norms.

Fourthly, consistent values and good trust relationships are the internal driving forces for professionals to actively and regularly participate in the supply of integrated chronic disease management services. In recent years, the influence of informal institutions on the integration of healthcare system, such as values, beliefs, social culture, and so forth, has gradually become a research hotspot. Some researchers have even proposed a specialized theoretical framework for this element [40]. Researchers have generally reached a consensus that the consistency of values, the belief in the benefits of inter-professional collaboration as well as the interpersonal trust are the internal driving factors that strongly influence the collaboration behaviors, interaction, and decision-making of different professionals [15,36,38,41,42,43,44,45,46]. Furthermore, the professionals’ identification of shared values was improved through a large number of propaganda and incentive measures in Youxi County. This resulted in the internalization of the external incentives, leading to professionals’ behaviors shift from passive collaboration to active and voluntary collaboration, hence considerably reducing the obstacles of professional integration so as to make the collaboration sustainable. This is what most regions of China and low- and middle- income countries with relatively backward economic and social development level lack. It is necessary for these countries and regions to pay more attention to the informal institutions while improving the formal ones, especially the formation of values such as people-oriented, prevention first, and so forth. On the other hand, due to the relatively poor service capability of the professionals in Youxi County’s primary healthcare institutions, the cognitive level of those in county hospitals on the benefits of inter-professional collaboration was lower than that of those in township hospitals and village clinics as well, thus this asymmetry of trust affected the implementation effects of the downward referral, which also offers new evidence to the existing research results.

### (4.2) The external conditions for ensuring professional integration of chronic disease management services

Our previous research shows that the professional integration at the micro level is affected by the external environment such as the system (government) at the macro level and the organization at the meso level, thus forming the empowerment and transmission chain of “system (government) -organization-individual.” [16,23,47]

Firstly, the fact that the government serves as an institutional supplier is an important guarantee for the professional integration of chronic disease management services. It is a significant prerequisite and guarantee for the smooth progress and success of reforms in every country that the government leaders attach a great importance and play a leading role in the institutional supply in order to create a favorable institutional environment [46,48]. As a demonstration area of health care system reform in China, Youxi County was appropriately empowered by governments of higher levels and had a high degree of autonomy in reform, which greatly enhanced its motivation and capacity to innovate the chronic disease management model. The main leaders of the county party committee and county government attached a great importance to the chronic disease management and were personally responsible for it, ensuring the rapid and orderly progress of this work. A multi-departmental collaboration mechanism was established to reduce the possibility of institution supply dilemma that might be due to departmental conflict of interests. A series of policies for chronic disease management were issued and the professionals’ opinions were incorporated into the process of policy making, which improved the scientificity and suitability of the policies. In addition, the government increased investment, providing financial support for chronic disease management. All the above experience is worth learning for other regions of China as well as the low- and middle- income countries with similar administrative systems. However, the sustainability of financial investment demands further attention.

Secondly, the tight county healthcare alliance lays an organizational foundation for professional integration of chronic disease management services. At present, the multi-sited practice policy of doctors has not been effectively implemented and widely promoted in China. The majority of professionals still need to provide services in their healthcare institutions. Therefore, the collaboration among professionals in different healthcare institutions is largely affected by the collaboration at the institutional level. The functions of healthcare institutions of different levels were redefined owing to the establishment of County General Hospital with a single legal representative in Youxi County, leading to the institutions’ behaviors shift from the original homogeneous competition to differentiated and synergetic development. The division of responsibilities of the professionals involved in chronic disease management service was just determined based on the functions of the institutions. Compared to the time before this reform, long-term collaboration mechanisms were established among institutions and more attention was paid to informatization construction and information interconnection, which not only assisted professionals in primary healthcare institutions to improve their capability of chronic disease management with the help of those in county hospitals, but also enhanced the trust among them in the long-term stable and efficient collaboration. Some research in China shows that the collaboration among different professionals is often much closer in areas with greater efforts to construct tight county healthcare alliances and better effects, and it is also more conducive to the development of integrated chronic disease management services [34,49,50]. This is not only consistent with our research findings, but also suggests that the collaboration at the institutional level could be strengthened through the establishment of tight healthcare alliance, healthcare groups and other forms in other regions of China and low – and middle-income countries with similar physician practice policies, so as to better promote the collaboration of chronic disease management among professionals in different institutions.

### (4.3) Innovations and limitations

This study conducted a case study on the “classified, color-coded, hierarchical and regionalized” chronic disease management model in Youxi County from the perspective of professional integration for the first time, which not only shows the latest practice of integrated chronic disease management services in China, but also provides another perspective for related research of chronic disease management. Additionally, the qualitative research is also conducive to a more comprehensive and an in-depth analysis of the case.

However, this study also has limitations. The property of a qualitative case study determines that the findings of this study cannot be generalized to other settings, whereas other regions of China as well as other low- and middle-income countries could still benefit from the lessons indicated in this case study. Besides, this study mainly conducts an in-depth analysis of the case from the dimension of professional integration, and lacks a more comprehensive analysis from other dimensions of integration such as system, organizational and care. Therefore, we should continuously keep track of the practice progress in Youxi County, conduct a comprehensive analysis of the case from every dimension of integration, and a mixed design combining quantitative and qualitative studies should be carried out to assess the practical effect and analyze the cause-and-effect association between the measures and effects in the future in order to provide further empirical evidence.

## (5) Conclusion

The “classified, color-coded, hierarchical and regionalized” chronic disease management model in Youxi County of Fujian Province is a beneficial exploration and innovative practice of an integrated chronic disease management service model on the basis of county healthcare alliances reform in the pioneer areas of health care system reform in China. Under the governmental institutional supply and the organizational support of the county healthcare alliances in Youxi County, a series of measures were taken, such as establishing the professional division and collaboration mechanisms, strengthening incentives and restraints, formulating unified inter-professional services norms and standards, and improving the compatibility of values and culture among different professionals. The practical experience of Youxi County can be replicated and promoted in other regions of China as well as the low – and middle-income countries with similar economic and social development levels, administrative systems and health management policies. However, the practice is in its early stage. There are still a lot of problems to be resolved and targeted measures should be taken, such as improving the chronic disease management service capacity of the primary healthcare institutions, perfecting the incentive and restraint mechanisms of professional collaboration, establishing the supervision, reward and punishment mechanisms to ensure the implementation of the inter-professional service norms and standards, and so forth. Moreover, in the future, a long-term, large-scale, quantitative and qualitative evaluation study on the effect of this case should be carried out in order to enrich the current research.
